# Genome-Wide Identification of the *GmATG* Gene Family and Its Response to Multiple Biotic and Abiotic Stresses in Soybean (*Glycine max*)

**DOI:** 10.3390/genes17090996

**Published:** 2026-08-24

**Authors:** Ling Yang, Jingyi Fan, Enguang Ren, Shuo Yang, Dandan Hu

**Affiliations:** Collaborative Innovation Center of Henan Grain Crops, College of Agronomy, Henan Agricultural University, Zhengzhou 450002, China; yl051115@163.com (L.Y.); 15238745979@163.com (J.F.); 18236769471@163.com (E.R.); 18837649597@163.com (S.Y.)

**Keywords:** soybean, autophagy, ATG genes, gene family evolution, biotic and abiotic stress

## Abstract

Background: Autophagy plays a central role in maintaining cellular homeostasis, regulating growth and development, and responding to multiple stresses. Autophagy-related genes (ATGs) play critical roles in autophagy, yet their functional diversity in soybean (*Glycine max*) remains underexplored. Methods: Genome-wide identification of *GmATG* genes was performed using sequence similarity and domain-based searches against the Wm82.gnm4 reference genome, followed by characterization of physicochemical properties, chromosomal distribution, phylogenetic relationships, gene duplication, conserved motifs, gene structure, three-dimensional structural, and promoter cis-acting elements. Tissue-specific expression and multiple stresses response were examined using transcriptome data and profiled by RT-qPCR. Results: A total of 60 *GmATG* genes belonging to 20 subfamilies were identified in soybean. Gene family expansion was predominantly driven by fragment duplication (33 gene pairs), with the *ATG8* family expanding to 12 members, and pan-genomic analysis uncovered prominent copy number variation (6–9 copies) in the *ATG18* family. *GmATG* genes showed distinct expression patterns in response to multiple abiotic and biotic stresses. Specifically, *GmATG18f* was significantly induced by phosphorus deficiency in the low-phosphorus-tolerant soybean variety Nannong 94-156. *GmATG8g*, *GmATG9d* and *GmATG13d* showed a typical expression trend of initial increase followed by decrease, with expression levels peaking at 6–12 h after salt stress treatment. *GmATG8g* and *GmATG9d* were rapidly upregulated at the early drought stress stage, while *GmATG13a* maintained sustained upregulation. In response to *Phomopsis* stem rot, *GmATG7a*/*8h*/*8i*/*11*/*13d*/*18e*/*18f* displayed differential expression in resistant and susceptible soybean materials. Conclusions: This study systematically characterizes the composition, expansion and stress response patterns of the *GmATG* gene family, revealing functional differentiation among family members. The identified key candidate genes, including abiotic-stress-regulated *GmATG8g*/*9d*/*13d*/*18f* and biotic-stress-regulated *GmATG7a*/*8h*/*8i*/*11*/*13d*/*18e*/*18f*, provide valuable genetic resources for the molecular breeding of stress-tolerant soybean.

## 1. Introduction

Autophagy is a highly conserved intracellular degradation pathway in eukaryotes. It involves the formation of double-membrane structures called autophagosomes, which encapsulate damaged organelles, abnormal protein aggregates, or exogenous pathogens within the cell. These autophagosomes fuse with vacuoles or lysosomes to complete degradation and material recycling. Autophagy plays a central role in maintaining cellular homeostasis, regulating growth and development, and responding to environmental stress [1,2,3]. This process is coordinately regulated by proteins encoded by autophagy-related genes (*ATG*s). ATGs were first identified through yeast genetic screens and comprised 32 members [4]. Subsequent research expanded this number to over 40, of which approximately 20 constitute the core autophagy machinery, whose function is highly conserved across yeast, animals, and plants [1]. These core ATG proteins form four major functional modules: kinase complexes, PI3K complexes, membrane recruitment complexes, and ubiquitin-like binding systems [1,5].

Unlike the single-copy pattern observed in yeast, the plant *ATG* gene family has undergone significant expansion during evolution. The expansion exhibits distinct modular characteristics: genes encoding the core catalytic enzymes (such as *ATG7*) are maintained as single copies to ensure the stability of the pathway’s fundamental functions, whereas gene families involved in substrate recognition and membrane recruitment (such as *ATG8* and *ATG18*) have experienced substantial expansion [6,7,8,9]. Moreover, the functions of *ATG* family members have undergone specialization: *ATG8a* is capable of simultaneously rescuing both carbon and nitrogen deprivation phenotypes, whereas *ATG8h* can only restore the carbon deprivation phenotype [10]; Maize (*Zea mays*) *ZmATG18a* specifically responds to drought stress [11]. At the mechanistic level, this functional differentiation is precisely regulated through post-translational modifications and substrate recognition. The E3 ubiquitin ligase seven in Absentia of *Arabidopsis thaliana* (SINAT) family dynamically regulates the stability of the ATG1–ATG13 kinase complex, thereby enabling precise switching between autophagy initiation and termination [12]. Furthermore, the substrate recognition mechanisms of selective autophagy also determine the functional specificity of autophagy. As a chloroplast autophagy receptor, Next to BRCA1 gene 1 (NBR1) specifically recognizes the ubiquitinated translocon at the outer envelope membrane of chloroplasts (TOC) complex and regulates chloroplast protein transport and stress tolerance via an ATG8-dependent pathway [3].

Autophagy plays an important role in plant responses to both biotic and abiotic stresses. Autophagy participates in salt adaptation by removing oxidized proteins and promoting Na^+^ compartmentalization [13]; *atg5* and *atg7* mutants are sensitive to salt stress, oxidative stress, and nutrient starvation [14], overexpression of rice (*Oryza sativa*) *OsATG8d*, *OsATG5* and *OsATG12* in *Escherichia coli* significantly improve *E. coli*’s growth under salt stress [15]. Autophagy participates in drought adaptation by regulating reactive oxygen species homeostasis (*ATG1* and *ATG8*) and modulating ABA signaling stress (*ATG1*) [16,17]. Under heat stress, *ATG8* enhances thermotolerance [18] and *ATG6* contributes to heat stress recovery [19]. Under nutrient stress, *OsATG8a* improves nitrogen uptake efficiency and grain yield [20], *OsATG8b* promotes nitrogen remobilization to seeds [21], *ATG6* can coordinate nitrogen uptake efficiency, carbon assimilation and nutrient remobilization in tomato [22], and *ATG8f* and *ATG8h* were induced under phosphorus deficiency [23,24]. In addition to abiotic stress, autophagy also plays pivotal roles in plant immunity [25]. During pathogen infection, autophagy can restrict the proliferation of biotrophic pathogens by promoting host cell death [26], while it may also suppress defense responses against necrotrophic pathogens to prevent excessive cell damage [27]. The immunomodulatory function of *ATG6* was identified in resistance to tobacco mosaic virus (TMV), pepper mild mottle virus, barley yellow dwarf virus, rice stripe mosaic virus, citrus yellow vein clearing virus, tomato spotted wilt orthotospovirus, potato virus Y, powdery mildew, clubroot, and southern corn rust [28,29]. *ATG8* was found to be involved in fine-tuning plant immunity [30]. The dual-role ATG1–ATG13 complex was found to act as a crucial switch in initiating or inhibiting autophagy in rice-gall-dwarf-virus-infected *Recilia dorsalis* [31].

Soybean (*Glycine max*) is an economically important crop supplying oil and protein. Previous studies have revealed that soybean *GmATG* genes exert divergent functions in response to distinct stress types. Under abiotic stress, nitrogen deficiency markedly upregulates the expression of *GmATG8c*, which modulates leaf senescence and internal nitrogen remobilization [32,33]. Under biotic stress, the silencing of core autophagy genes *GmATG2* or *GmATG7* accelerates senescence and enhances resistance to bacterial and viral pathogens [34,35], indicating that autophagy negatively regulates soybean immunity responses. Despite these findings, comprehensive understanding of the soybean *GmATG* gene family remains limited due to substantial research gaps. As a paleotetraploid species shaped by two independent whole-genome duplication (WGD) events [36], the evolutionary mechanisms underlying *GmATG* family expansion, the respective contributions of segmental and tandem duplication to gene proliferation, and the correlation between duplication patterns and functional divergence of paralogous *GmATG* genes remain largely unclear. In addition, current studies only focus on sporadic individual *GmATG* members, lacking systematic genome-wide profiling of *GmATG* transcriptional landscapes and their regulatory associations with plant response to salt, drought, phosphorus deficiency, and pathogen challenges. To fill these research gaps, the present study adopted comprehensive bioinformatics approaches to systematically characterize the *GmATG* gene family in soybean. We systematically analyzed the gene structure, conserved domains, chromosomal distribution, and duplication patterns of all identified *GmATG* members. Pan-genome data were integrated to explore gene copy number variation, and single-cell transcriptome datasets were used to dissect the tissue-specific expression heterogeneity of the *GmATG* family. Furthermore, we comprehensively profiled the transcriptional responses of *GmATG* genes to multiple stresses conditions, including phosphorus deficiency, salinity, drought, and *Phomopsis longicolla* infection. Collectively, our findings clarify the structural characteristics and evolutionary expansion mechanisms of the *GmATG* gene family and screen out several core stress-responsive candidate genes, such as *GmATG18f* and *GmATG8h*. This study provides valuable genetic resources and a solid theoretical basis for further elucidating the molecular regulatory mechanisms of autophagy underlying stress adaptation in legumes and facilitates the molecular breeding of stress-tolerant soybean varieties.

## 2. Materials and Methods

### 2.1. Identification of ATG Genes in Soybean

To obtain query sequences for homology searches, protein sequences were first retrieved from published lists of *Arabidopsis thaliana ATG* (*AtATG*) genes [8] and rice *ATG* (*OsATG*) genes [9]. The ATG amino acid sequences of *A. thaliana*, rice and soybean were downloaded from TAIR (https://www.arabidopsis.org/; accessed on 17 October 2025), Ensembl Plants (https://plants.ensembl.org/; accessed on 17 October 2025), and SoyBase (https://www.soybase.org/; accessed on 17 October 2025) [37], respectively (Table S1).

A two-pronged strategy was adopted to identify members of the *GmATG* gene family. First, a homology analysis was carried out using TBtools (v2.400) [38]: local BLASTP searches were conducted against the soybean protein database, using AtATG and OsATG protein sequences as queries, with an E-value threshold of 1 × 10^−5^. Second, a hidden Markov model (HMM) search was performed. HMMs of autophagy-related protein families (derived from the Pfam database, including ATG1 (PF04497), ATG2_CAD (PF13329), ATG4_LIR (PF20166), ATG8 (PF02991), ATG9 (PF04111), etc.) were downloaded from the InterPro database (https://www.ebi.ac.uk/interpro/; accessed on 17 October 2025). The soybean proteome was scanned using the hmmsearch program in HMMER 3.3, with an E-value threshold set to 1 × 10^−5^ [39]. The candidate protein IDs obtained from the BLASTP and HMM searches were merged, and redundant sequences were removed to generate a preliminary list of candidate genes. Finally, SMART (http://smart.embl-heidelberg.de/; accessed on 22 October 2025) and NCBI CDD (https://www.ncbi.nlm.nih.gov/Structure/cdd/wrpsb.cgi/; accessed on 22 October 2025) [40] online tools were used to validate the domains of candidate proteins, retaining only those containing complete and typical ATG domains as the final members *of* the *GmATG* gene family.

### 2.2. Analysis of the Basic Physicochemical Properties of the GmATG Proteins

The basic physicochemical properties of each GmATG protein, including the number of amino acids, molecular weight, theoretical isoelectric point (pI), and grand average of hydropathicity (GRAVY), were analyzed using the ExPASy ProtParam tool (https://web.expasy.org/protparam/; accessed on 6 November 2025). Subcellular localization of all GmATG proteins was predicted using the Plant-mPLoc server (http://www.csbio.sjtu.edu.cn/bioinf/plant-multi/; accessed on 6 November 2025) and WoLF PSORT (https://wolfpsort.hgc.jp/; accessed on 6 November 2025). When both tools yielded consistent predictions, the result was adopted as the final assignment. In cases of discrepancy, the WoLF PSORT prediction was prioritized, as this tool has been shown to have higher overall accuracy for plant protein localization (approximately 86%) compared to Plant-mPLoc (approximately 64%) in independent benchmark studies [41,42]. The predicted localizations are presented in Table S2.

### 2.3. Phylogenetic Analysis and Nomenclature of the GmATG Family

To clarify the phylogenetic relationships and classify *GmATG* genes, reference sequences of reported *Arabidopsis* and rice ATG proteins were obtained from TAIR (https://www.arabidopsis.org/; accessed on 10 November 2025) and Ensembl Plants (https://plants.ensembl.org/; accessed on 10 November 2025). Multiple sequence alignment was performed using the MUSCLE module in TBtools (v2.400). Based on the alignment results, a maximum likelihood (ML) phylogenetic tree was constructed using IQ-TREE(v2.2.0) [43], with automatic model selection and 1000 bootstrap replicates to assess branch support. Branch support was interpreted following the conventions of plant phylogenetic studies: bootstrap values above 90% were considered highly supported; values between 70% and 90% were considered moderately supported; values between 50% and 70% were considered weakly supported and interpreted with caution; and values below 50% were considered unsupported and are not shown in the final tree. The generated tree file was imported into the iTOL (v7) online platform (https://itol.embl.de/; accessed on 11 November 2025) for visualization and adjustment. Based on the clustering relationships of soybean genes with their homologs in *Arabidopsis* and rice on the phylogenetic tree, the soybean genes were classified into the corresponding ATG protein subfamilies. The complete list of *GmATG* genes, their names, systematic identifiers, and subfamily affiliations are detailed in Table S2.

### 2.4. Chromosomal Localization, Duplication Events, and Collinearity Analysis of GmATG Family

Based on chromosome and gene location data obtained from the soybean genome database SoyBase (https://www.soybase.org/; accessed on 20 November 2025), systematic genomic and evolutionary analyses of the *GmATG* gene family were performed using TBtools (v2.400) [38]. Whole-genome collinearity analysis within soybean was performed using the “One Step MCScanX” tool integrated into TBtools. The software parameters were set as follows: a BLASTP E-value threshold of 1 × 10^−10^, retaining the top 5 best matches for each gene. Subsequently, the “File Merge For MCScanX” tool was used to filter the preliminary results, with a minimum block size set to 5 to eliminate random matches. Based on this, homologous *GmATG* gene pairs located within the aforementioned collinearity blocks were defined as fragment duplication events, while adjacent homologous gene pairs with a physical distance of less than 10 kb were defined as tandem duplication events.

For interspecies comparisons, genome sequences and annotation files for *Arabidopsis thaliana* and wild soybean (*Glycine soja*) were retrieved from TAIR (https://www.arabidopsis.org/; accessed on 24 November 2025) [44] and from the Ensembl Plants genome database (https://plants.ensembl.org/; accessed on 24 November 2025) [45]. The “One Step MCScanX” tool (with the same parameters as above) was used to calculate whole-genome synteny between soybean and *Arabidopsis* as well as between soybean and wild soybean, filtering results with a minimum block size of 5. All results (including intraspecific gene distribution, duplication events, and interspecific collinearity) were ultimately integrated and visualized using the “Advanced Circos” module of TBtools. To systematically present the collinear relationships of *GmATG* genes, we compiled Table S3, which lists all collinear gene pairs, including orthologous relationships between soybean and *A. thaliana*, and between soybean and wild soybean, as well as paralogous relationships among soybean genes identified through segmental duplication. Functional annotations in this table are primarily derived from the experimentally validated *A. thaliana* orthologs, sourced from the TAIR database, with references provided as DOIs. For wild soybean orthologs, functional annotations were not independently assigned; they were inferred directly from the corresponding soybean genes based on sequence identity and synteny. Genes for which no syntenic ortholog was detected in the soybean–*A. thaliana* collinear blocks (not listed in this table) are likely due to species-specific expansion or lineage-specific loss.

### 2.5. Pan-Genomic Analysis of the GmATG Family

To investigate copy number variations (CNVs) of *GmATG* genes across soybean populations, we retrieved the presence/absence data for the 60 *GmATG* genes in 29 soybean accessions from the SoyOmics database (https://ngdc.cncb.ac.cn/soyomics/index; accessed on 11 December 2025) [46,47]. These accessions included 16 cultivars (W82 (Williams 82), ZH13 (Zhonghuang 13), and SoyC01–SoyC14), 9 landraces (SoyL01–SoyL09), and 4 wild soybeans (W05, SoyW01–SoyW03). The SoyOmics database provides presence/absence calls for each gene across the 29 accessions based on whole-genome sequence comparisons against the reference genome (W82). For each *GmATG* gene, the corresponding orthologous gene ID(s) in each accession are listed where present (Table S4). In cases where one reference gene matches multiple sequences in an accession, all significant orthologs are listed, reflecting the occurrence of gene duplication events. We extracted the presence/absence matrix for all 60 *GmATG* genes and counted the number of genes present in each accession for each ATG subfamily to determine the copy number of that subfamily (Table S5). Genes detected in all 29 accessions were classified as core genes, while those absent in at least one accession were classified as variable genes. Based on the copy number statistics described above, the distribution of copy numbers for each *ATG* subfamily across the 29 accessions was plotted using GraphPad Prism 10.

### 2.6. Analysis of Conserved Motifs, Domains, Gene Structures, and 3D Protein Structures of the GmATG Family

Conserved motif identification of GmATG protein sequences was performed using MEME Suite (v5.5.9) (https://meme-suite.org; accessed on 20 January 2026) [48], with the maximum number of motifs set to 15 to ensure comprehensive coverage of all potential discrete conserved patterns within the family. Subsequently, the conserved domains were annotated using NCBI Batch CD-Search (https://www.ncbi.nlm.nih.gov/cdd; accessed on 20 January 2026) [40]. Gene structural information (exon–intron distribution) was obtained from the soybean genome database SoyBase (https://www.soybase.org/; accessed on 17 October 2025) and visualized using the “Gene Structure View (Advanced)” module in TBtools (v2.400) [38]. The intron phase of *GmATG* genes is shown in Table S6. Homology modeling of representative GmATG proteins was performed using the SWISS-MODEL server (https://swissmodel.expasy.org; accessed on 22 January 2025) [49]. The 3D models of the target proteins were constructed using the known structure with the highest sequence similarity as a template, selected via the automatic template identification system. For each predicted model, the Global Model Quality Estimation (GMQE) and sequence identity (Seq Identity) were calculated. Considering that some models exhibited high sequence identity with the template (up to 100%), they were included in subsequent comparative analyses even though their GMQE was slightly below 0.75. Based on the clustering relationships in the phylogenetic tree, three-dimensional structures were compared both within the same subfamily and across different subfamilies.

### 2.7. Analysis of cis-Acting Elements in GmATG Gene Promoters

First, genomic sequences spanning 2000 bp upstream of the start codon of the *GmATG* gene were retrieved and extracted from the SoyBase database (https://www.soybase.org/; accessed on 7 January 2026) [37]. These regions were defined as potential promoter regions. Subsequently, the PlantCARE online tool (http://bioinformatics.psb.ugent.be/webtools/plantcare/html/; accessed on 7 January 2025) [50] was used to analyze these promoter sequences to predict the *cis*-acting elements they contain. Finally, the PlantCARE prediction results were organized and visualized using the TBtools software (v2.400), generating a distribution map of *cis*-acting elements within the promoter sequences to facilitate comparison of the types, numbers, and arrangement patterns of promoter elements among different *ATG* genes.

### 2.8. Analysis of Expression Characteristics of the GmATG Family Based on Transcriptomics Data

To systematically analyze the expression patterns of *GmATG* genes, transcriptomic data from the SoyOmics database (https://ngdc.cncb.ac.cn/soyomics/transcriptome/tissues; accessed on 17 January 2026) [51] were used to analyze the expression profiles of this gene family across different developmental stages and tissues of the W82 variety (Table S7). Samples included roots, cotyledons, leaves, stems, flowers, pods, and seeds from the emergence stage (VE5), cotyledon stage (VC), three-node stage (V3), flowering stage, and various stages of seed development. After obtaining the FPKM values for the genes, they were normalized using log_2_ transformation, and expression heatmaps were generated using TBtools (v2.400).

To further elucidate the spatial heterogeneity of gene expression at the cellular level, two independent single-cell transcriptomics databases were analyzed in depth. First, single-cell RNA sequencing data of W82 were obtained from the Soybean Multi-Omic Atlas database (https://soybean-atlas.com/; accessed on 20 January 2026) [52], and single-cell transcriptome data of ZH13 (root, stem, leaf, and root nodule) were obtained from the SoyOmics database (https://ngdc.cncb.ac.cn/soyomics/index; accessed on 20 January 2026) [51]. The expression patterns of *GmATG* genes in different cell types were retrieved from these databases and compiled for presentation. For ZH13, nodules, roots, stems, leaves, and shoot apical meristems were collected at the vegetative cotyledon stage; nodules were harvested 5 days after inoculation with *Bradyrhizobium* USDA-110.

Analysis of existing transcriptomic data: Previously published transcriptomic data of leaves and roots under low phosphorus stress for 14 d [53], leaves and roots under salt stress for 6 h [54], and roots under drought stress for 6 d [55] were used to analyze the expression profiles of *GmATG* genes under abiotic stress. The salt stress dataset [54] and drought stress dataset [55] were deposited in the NCBI Gene Expression Omnibus (GEO) under accession numbers GSE173640 and GSE40627, respectively.

The transcriptomic data of stems of four soybean lines (susceptible lines H197 and H027; resistant lines H229 and H267) after infection with *P. longicolla* N34 (unpublished) were used to analyze the expression profiles of *GmATG* genes under biotic stress. Seedlings of soybeans were grown in boxes filled with nutrient soil and loess (1:2) for 14–16 days under controlled growth chamber conditions (26–28 °C, 14 h photoperiod). Soybean seedlings were cut from the first true leaves with a sharp knife, the transverse section of the soybean stem was covered with a yellow spear head (200 mL) containing *P. longicolla* N34, then soybean seedlings were grown in an artificial climate chamber with a temperature of 30 °C and a humidity of 80%. Samples were taken from the cotyledonary node to the upper stem of the soybean 1 d after inoculation and stored in a −80 °C freezer. Three plants were taken as three biological replicates of each soybean to perform transcriptomic sequencing. The RNA extraction, library construction, and sequencing operations were completed by Guangdong Jidiao Biotechnology Co., Ltd. (Guangzhou, China) on a Illumina novaseq 6000 sequencing platform. And the data analysis was performed as reported in Hu et al. [53].

The input data formats for heatmap generation differed across studies. For the low-phosphorus (Table S8) and *P. longicolla* datasets (Table S9), expression matrices (FPKM or log_2_-FPKM) were used. For the drought (Table S10) and salt stress datasets (Table S11), we extracted log_2_FC, *p*-value, and FDR values of differentially expressed genes identified by the original authors (thresholds: *p* < 0.05 and FDR < 0.05) and selected those belonging to the *GmATG* family. Only genes that reached the significance thresholds under the corresponding stress conditions were shown in heatmaps. All values were subjected to row-wise Z-score normalization for heatmap visualization. Thus, the heatmaps for low-phosphorus and pathogen infection display expression levels, whereas those for drought and salt stress display fold-change responses. Since all data were obtained as pre-normalized results, no additional cross-dataset normalization or batch effect correction was applied. Complete lists of *GmATG* genes significantly responding to drought and salt stresses are provided in Tables S9 and S10, and the expression matrices for low-phosphorus and *P. longicolla* datasets are provided in Tables S7 and S8.

### 2.9. Plant Materials and Stress Treatments

For all stress treatments, seeds were surface-sterilized with chlorine gas for 5–6 h, and residual chlorine was dissipated in an ultra-clean bench for approximately 30 min. Sterilized seeds were germinated in autoclaved vermiculite under controlled growth chamber conditions (28–33 °C day/20–25 °C night, 14 h photoperiod). Five days after sowing, uniformly germinated seedlings were transferred to hydroponic culture in half-strength Hoagland solution (as described in [53]) with the nutrient solution replaced every 2 days. Seedlings were grown until the V1 stage (when the first trifoliate leaf was fully expanded) before stress treatments were applied. Salt stress treatment: V1-stage seedlings of W82 were transferred to 1/2 Hoagland medium supplemented with 120 mM NaCl to induce salt stress. Control plants were treated with an equimolar concentration of KCl (120 mM) to maintain isotonic conditions. This design follows the commonly used method in previous salt stress studies to distinguish between ionic toxicity and osmotic effects [56]. After 0 h, 3 h, 6 h, 12 h, 1 d, 2 d, and 3 d of treatment, root tissues were collected with three biological replicates, immediately frozen in liquid nitrogen, and stored at −80 °C for subsequent RNA extraction.

Drought stress treatment: V1-stage seedlings were transferred to hydroponic solution containing 20% polyethylene glycol 6000 (PEG-6000); the control group was treated with PEG-free hydroponic solution. At 0 h, 3 h, 6 h, 12 h, 1 d, and 2 d after treatment, root tissues were collected with three biological replicates, immediately frozen in liquid nitrogen, and stored at −80 °C for subsequent RNA extraction.

Low-phosphorus stress treatment: At the V2 stage (when the second trifoliate leaf was fully expanded), the low-phosphorus-tolerant soybean variety Nannong 94-156 (NN94-156) and the sensitive variety Bogao were selected and cultivated under standardized conditions in a controlled-environment climate chamber. Plants were transferred to hydroponic culture in 1/2-strength modified Hoagland nutrient solution containing either normal phosphorus (NP: 500 µM KH_2_PO_4_) or low phosphorus (LP: 5 µM KH_2_PO_4_ + 495 µM KCl to maintain ionic balance). The nutrient solution composition was based on [53]. After 11 days of hydroponic cultivation, root tissues were collected with three biological replicates, flash-frozen in liquid nitrogen, and stored at −80 °C for RNA extraction.

### 2.10. RNA Extraction and Reverse Transcription Quantitative PCR (RT-qPCR) Analysis

According to the manufacturer’s instructions, total RNA was extracted from the roots or leaves of each treatment group using the SPARKeasy Plant RNA Extraction Kit (SparkJade, Qingdao, China). RNA concentration and purity were assessed using a NanoDrop 2000 spectrophotometer (Thermo Fisher Scientific, Massachusetts, USA), and RNA integrity was verified via agarose gel electrophoresis. Using 1 µg of total RNA, cDNA was synthesized via reverse transcription using the SPARKscript II RT Plus Kit with gDNA Eraser (SparkJade, Qingdao, China). Using the cDNA as a template, RT-qPCR reactions were performed on the CFX96 Touch real-time quantitative PCR system (Bio-Rad Laboratories, Hercules, CA, USA). The reaction volume was 20 µL, containing 10 µL of 2× SYBR Green qPCR Mix (SparkJade, China), 200 nM of forward and reverse primers, and approximately 10 ng of cDNA template. The soybean *tubulin* gene (GenBank accession number: AY907703) was used as internal control. The reaction protocol was as follows: 30 s pre-denaturation at 95 °C; 5 s denaturation at 95 °C, 30 s annealing at 60 °C, for 40 cycles; following the reaction, a melting curve analysis was performed (65–95 °C, heating rate 0.5 °C/s) to verify primer specificity. Three technical replicates were set up for each sample. Relative gene expression levels were calculated using the 2^−ΔΔCt^ method [57]. The primer sequences used in this study are detailed in Table S12.

## 3. Results

### 3.1. Identification and Analysis of the Physicochemical Properties of the GmATG Family

**Figure 1:**
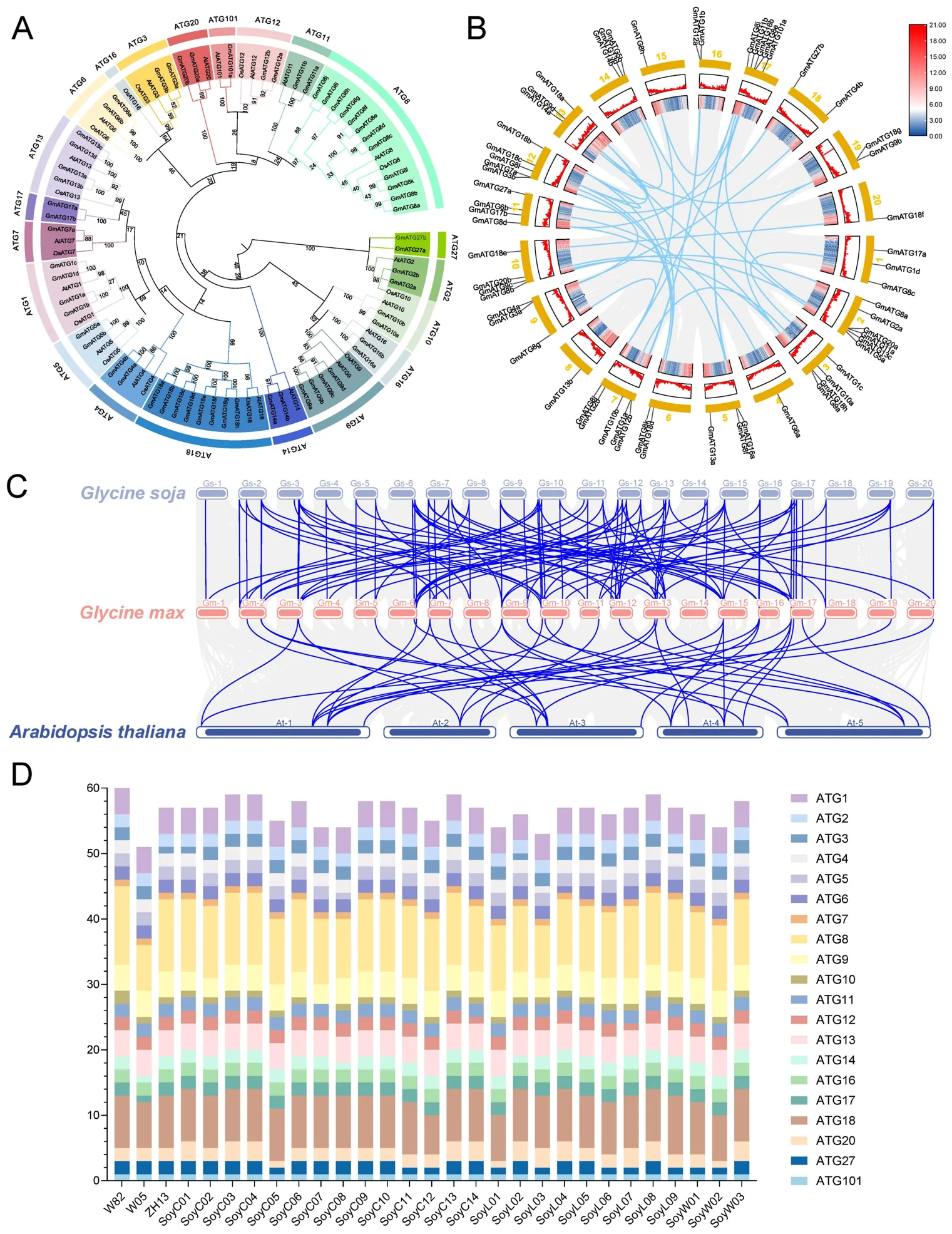
Identification and evolutionary analysis of the *GmATG* genes. (**A**) Maximum likelihood phylogenetic tree of ATG proteins from soybean (*Glycine max*, Gm), *Arabidopsis thaliana* (At), and rice (*Oryza sativa*, Os). The tree was constructed using IQ-TREE with 1000 bootstrap replicates. Different subfamilies are distinguished by different colors. (**B**) Intraspecific collinearity analysis in soybean. Blue lines connect *GmATG* gene pairs involved in segmental duplication events. (**C**) Interspecific collinearity analysis between soybean and *Arabidopsis* and between soybean and wild soybean (*Glycine soja*, Gs). Gray lines indicate genomic collinearity blocks; blue lines highlight orthologous gene pairs. (**D**) Pan-genomic analysis of copy number variation of *ATG* genes across 29 soybean accessions. The color gradient represents the presence frequency of each subfamily member among the accessions.

Through genome-wide screening, a total of 60 *GmATG* homologous genes were identified in the soybean genome (Table S1). Based on phylogenetic analysis, these genes were classified into 20 distinct *GmATG* subfamilies (Figure 1A), and the *GmATG* genes were named according to the subfamilies and the paralogous genes distinguished by letter suffixes, *such as GmATG1a-d*, *GmATG4a-b* (Table S1). Each *GmATG* subfamily exhibited considerable heterogeneity in amino acid length and molecular weight, the amino acid lengths ranged from 94 aa (GmATG12) to 1978 aa (GmATG2b) and the predicted molecular weight ranged from 10.51 kDa (GmATG12) to 217.16 kDa (GmATG2). Notably, the extreme members clearly corresponded to specific subfamilies, with the largest and smallest proteins belonging to the GmATG2 and GmATG12 subfamilies, respectively (Table S2). The theoretical isoelectric point (pI) distribution ranged widely, from 4.69 (GmATG5b, GmATG17b) to 9.25 (GmATG12), 27 GmATG proteins were alkaline (pI > 7.0) and 33 *GmATG* were acidic (pI < 7.0). The hydropathicity (GRAVY) value of GmATGs ranged from −0.573 (GmATG3b) to 0.316 (GmATG27b), with only five GmATGs having positive GRAVY values. Of the GmATG proteins, 60 were predicted widely distributed across multiple cellular compartments, including the cytoplasm (19), nucleus (19), chloroplast (12), plasma membrane (8), endoplasmic reticulum (1), and Golgi apparatus (1). Members within the same subfamily generally exhibited conserved localization patterns: all ATG4 and ATG20 members were localized to the chloroplast; all ATG2, ATG3, ATG10, ATG11, and ATG13 members localized to the nucleus; all ATG5, ATG6, and ATG12 members localized to the cytoplasm; and all ATG9 and ATG27 members localized to the plasma membrane. In contrast, ATG1, ATG8, ATG17, and ATG18 subfamilies showed two to three distinct localizations, with ATG18 displaying the most pronounced divergence (two members to the plasma membrane, six members to the chloroplast).

### 3.2. Phylogenetic and Evolutionary Analysis of the GmATG Family

To elucidate the phylogenetic relationships and potential functional divergence of *ATG* genes, amino acid sequences of ATGs from *Arabidopsis*, rice, and soybean were integrated to construct a maximum likelihood phylogenetic tree (Figure 1A). Among the 20 *ATG* subfamilies, the *ATG8* subfamily had the most *GmATG* genes (12, designated *GmATG8a–GmATG8l*). The phylogenetic topology indicated that the vast majority of ortholog genes from soybean, *Arabidopsis*, and rice were clustered within the same subfamily. However, a few exceptions were also observed: *OsATG6* belonged to the same *ATG6* group, but it did not form a subclade with its dicotyledonous homologs.

The *GmATG* genes were unevenly distributed across the 20 chromosomes of soybean (Figure 1B). Among them, chromosomes 2 and 7 hosted the most *GmATG* genes (six and five, respectively), while chromosomes 4, 8, 15, and 20 hosted the fewest (only one each). Collinearity analysis identified 33 pairs of homologous *GmATG* genes resulting from segmental duplication events, a number far exceeding that of tandem duplication events. Interspecies comparative analysis revealed extensive collinearity in the ATG locus region between soybean and wild soybean, with direct homologs for the vast majority of *GmATG* genes present in wild soybean (Figure 1C). In contrast, the number of collinearity blocks between soybean and *Arabidopsis* was significantly reduced. *ATG8* and *ATG18* subfamily genes exhibit extensive syntenic conservation across soybean, *A. thaliana*, and wild soybean, whereas several *GmATG* members—including *GmATG3a/b*, *GmATG4a/b*, *GmATG6a/b*, *GmATG7a*, *GmATG13c/d*, and *GmATG16a/b*—lack detectable *A. thaliana* orthologs, suggesting species-specific expansion or lineage-specific loss within the *Glycine* lineage (Table S3).

### 3.3. Copy Number Variation of the GmATG Family

To elucidate the genetic variation of *GmATG* genes in soybean natural populations, pan-genomic analysis was performed on 29 representative soybean accessions including 16 cultivars (W82, ZH13, and SoyC01–SoyC14), nine landraces (SoyL01–SoyL09), and four wild soybeans (W05, and SoyW01–SoyW03) (Figure 1D). The total number of *GmATG* genes across different accessions ranged from 51 (W05) to 60 (W82), and cultivars, landraces, and wild soybeans contained on average 57.13, 56.22, and 54.50 *GmATG* genes, respectively (Table S5). Among these, several subfamilies encoding core components of the autophagosome assembly exhibited strict copy number conservation across all accessions, suggesting the indispensability of these genes for core autophagy functions. Specifically, the copy numbers of *ATG2* (2), *ATG7* (1), *ATG11* (2), *ATG13* (4), *ATG16* (2), *and ATG101* (1) were identical across all 29 accessions. In contrast, the *ATG8* and *ATG18* subfamilies exhibited extensive copy number variation (CNV). Gene number of *ATG8* ranged from 7 (W05) to 12 (W82), while gene number of *ATG18* ranged from 6 (SoyC12) to 9 (SoyL09). Additionally, *GmATG8d* and *GmATG10b* were W82 specific (Table S4).

### 3.4. Analysis of Domains, Conserved Motifs, and Gene Structures in the GmATG Family

Soybean *GmATG* members could be classified into six groups based on their functional complex affiliations: the *ATG1*/*13* kinase complex (including *GmATG1*, *GmATG11*, *GmATG13*, *GmATG17*, and *GmATG101a*), the PI3K complex (including *GmATG6* and *GmATG14*), the *ATG9*/*2*/*18* complex (including *GmATG2*, *GmATG9*, and *GmATG18*), the ubiquitin-like *ATG8* and its PE-binding complex (including *GmATG3*, *GmATG4*, *GmATG7a*, and *GmATG8*), the ubiquitin-like *ATG12* and *ATG5*-binding complex (including *GmATG5*, *GmATG7a*, *GmATG10*, *GmATG12*, and *GmATG16*), and SNARE complexes (including *GmATG20* and *GmATG27*). The number of members per complex varied, with the ubiquitin-like *ATG8* and its PE-binding complex having the most members (17), while the SNARE complex had the fewest (4).

**Figure 2:**
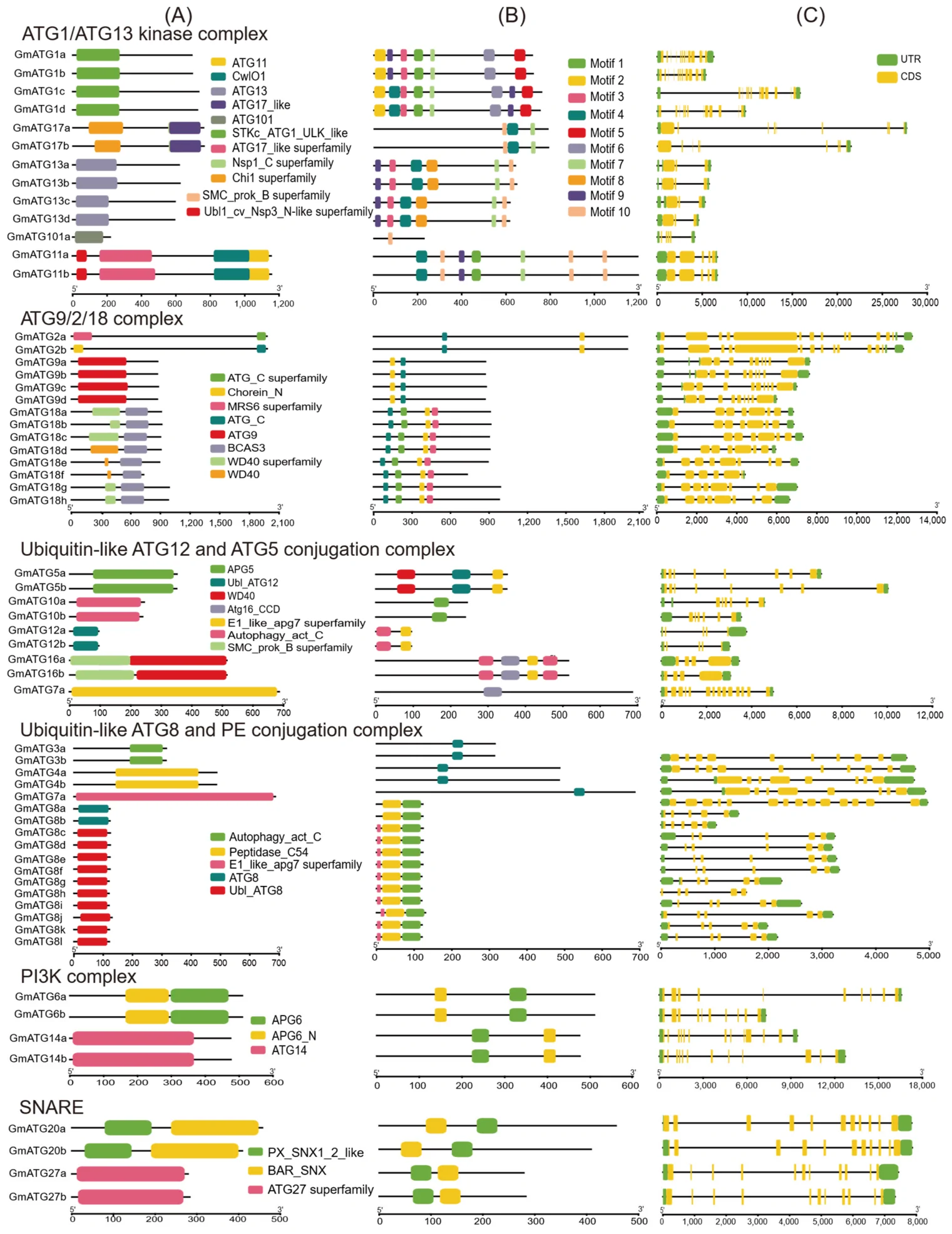
**Domain, conserved motif, and gene structure analysis of the *GmATG* family.** (**A**) Conserved domain composition of GmATG proteins. Different domains are represented by colored boxes. (**B**) Conserved motifs (Motif 1–15) predicted by MEME Suite. Different colors represent different motif types. (**C**) Gene structures of *GmATG* genes. Yellow boxes: exons (CDS); black lines: introns; green boxes: untranslated regions (UTRs).

Analysis of conserved domains indicated that members within the same functional complex shared highly similar domain compositions (Figure 2A). For example, members of the *GmATG1* kinase subfamily all contained a serine/threonine protein kinase domain (STKc_*ATG1*); *GmATG2a* contained MRS6 superfamily and *ATG_C* superfamily domains, whereas *GmATG2b* possessed Chorein_N and *ATG_C* domains. Within the *ATG8* subfamily, *GmATG8a* and *GmATG8b* contained typical *ATG8* domains, whereas *GmATG8c*-*GmATG8l* contained Ubl-ATG8 domains. In the *ATG18* subfamily, *GmATG18a*-*GmATG18d* and *GmATG18g*-*GmATG18h* contained WD40-superfamily domains, *GmATG18d-GmATG18f* contained WD40 domains, and all *ATG18* members contained BCAS3 domains. Domain conservation was observed across the other subfamilies.

Analysis of conserved motifs revealed significant differences in the average number of motifs among functional complexes (Figure 2B): the *ATG1*/*13* kinase complex, *ATG9*/*2*/*18* complexes, the ubiquitin-like *ATG12*-*ATG5* binding complex, the *ATG8*-PE conjugation complex, and the PI3K-SNARE complex contained 1~8, 2~4, 1~4, 1~3, and 2 motifs, respectively.

Analysis of gene structure revealed that the number of exons in the *GmATG* family exhibited a wide range of variation (3–14) and was closely associated with functional subfamilies (Figure 2C). Among members of the *ATG1*/*13* kinase complex, the *ATG1* family consisted of 13 exons, while the *ATG13* family consisted of three exons, representing the most simplified structure. Among the members of the *ATG2*/*9*/*18* membrane-recruiting complex, *GmATG2* subfamily has 13 exons, *GmATG9* subfamily has 9 exons, and the *GmATG18* subfamily has 7–9 exons. Members of the ubiquitin-like binding system exhibited a high degree of conservation within subfamilies: the *GmATG8* subfamily has five exons, among which *GmATG8h* was the only one lacking both a 5′ UTR and 3′ UTR. The PI3K complex, *GmATG6* and *GmATG14* subfamilies, had 10 and 12 exons, respectively, while the SNARE complex (*GmATG20* and *GmATG27* subfamilies) had 11 exons, demonstrating high conservation within the subfamilies. Intron phase analysis revealed that phase 0 introns were the most abundant across the *GmATG* gene family, accounting for approximately 70% of all introns. Phase 1 and phase 2 introns showed subfamily-specific distribution patterns, with phase 1 introns predominantly present in the *GmATG1* and *GmATG2* subfamilies, while phase 2 introns were relatively rare and mainly observed in the *GmATG18* subfamily. Notably, the majority of *GmATG* subfamilies exhibited identical intron phase patterns among their members, indicating strong evolutionary conservation of splice sites. However, the *GmATG6* and *GmATG18* subfamilies showed intrasubfamily divergence, suggesting potential functional differentiation or structural rearrangement within these specific subfamilies (Table S6).

### 3.5. Three-Dimensional Structural Analysis of GmATG Proteins

**Figure 3:**
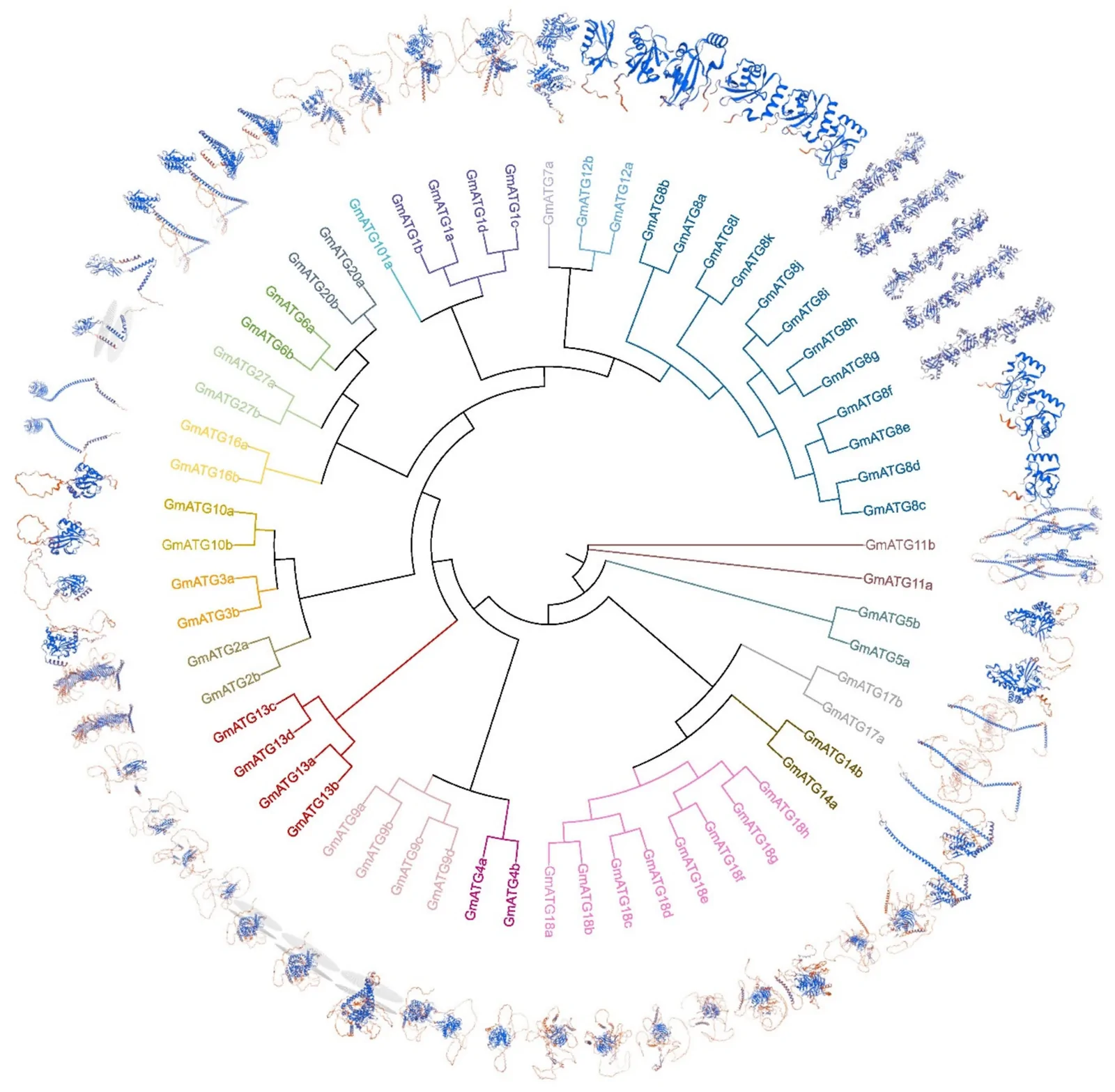
**Three-dimensional structural prediction of representative *GmATG* proteins.** Three-dimensional structures of GmATG proteins predicted by homology modeling using SWISS-MODEL. Branches of the phylogenetic tree are colored to distinguish different *GmATG* subfamilies, and the corresponding predicted 3D protein structures are displayed on the outer ring.

To elucidate the structural basis of GmATG proteins, homology modeling was performed using SWISS-MODEL, and a comparative analysis of the three-dimensional structures was conducted based on phylogenetic relationships (Figure 3). Overall, the three-dimensional structures of GmATG proteins exhibited significant diversity but showed high overall conservation within subfamilies. However, significant differences existed in the predicted structures among members of the GmATG8 subfamily. While GmATG8f-GmATG8j shared similar three-dimensional structures, they differed markedly from those of GmATG8a-GmATG8e and GmATG8k-GmATG8l. These members exhibited distinct characteristics in key aspects such as helical arrangements, loop orientations, and domain alignments.

### 3.6. Analysis of cis-Acting Elements in the GmATG Family

**Figure 4:**
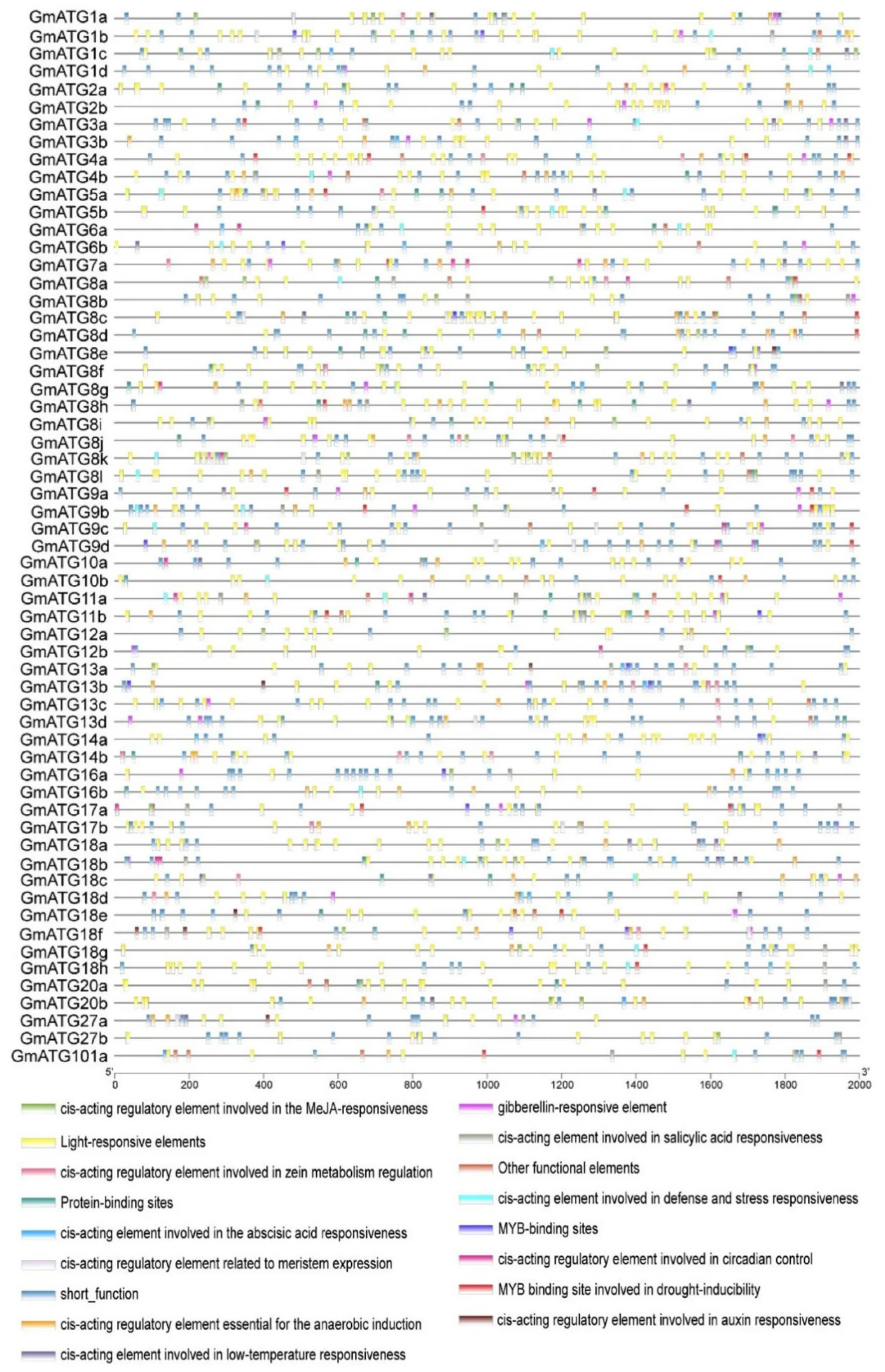
***Cis*-acting element analysis of *GmATG* gene promoters.** Distribution of *cis*-acting elements in the promoter regions (2000 bp upstream of the transcription start site) predicted by PlantCARE. Different colors indicate different functional categories.

To systematically elucidate the functional characteristics of the soybean *GmATG* gene family, promoter sequences spanning 2000 bp upstream of the start codon were extracted for each member of the family, and a systematic identification of *cis*-acting elements was performed using the PlantCARE database (Figure 4). The results showed that basic transcriptional regulatory elements (core promoter/enhancer elements) predominated in number, and the promoter regions of *GmATG* genes were rich in various environmental and hormonal response elements. Among these, light-responsive elements were the most abundant of all response element types. Regarding hormonal responses, response elements associated with abscisic acid (ABA), methyl jasmonate (MeJA), and gibberellin (GA) were significantly more numerous than those associated with auxin (IAA) and salicylic acid (SA). In addition, the promoter regions contained a wide distribution of regulatory sites associated with abiotic stresses such as drought, cold, and hypoxia and included specific MYB transcription factor binding sites. In contrast, the number of regulatory elements associated with specific biological processes (such as alcohol-soluble protein metabolism, meristematic tissue expression, and circadian rhythm control) was relatively low.

### 3.7. Analysis of the Tissue Expression Profile of GmATG Gene Family

**Figure 5:**
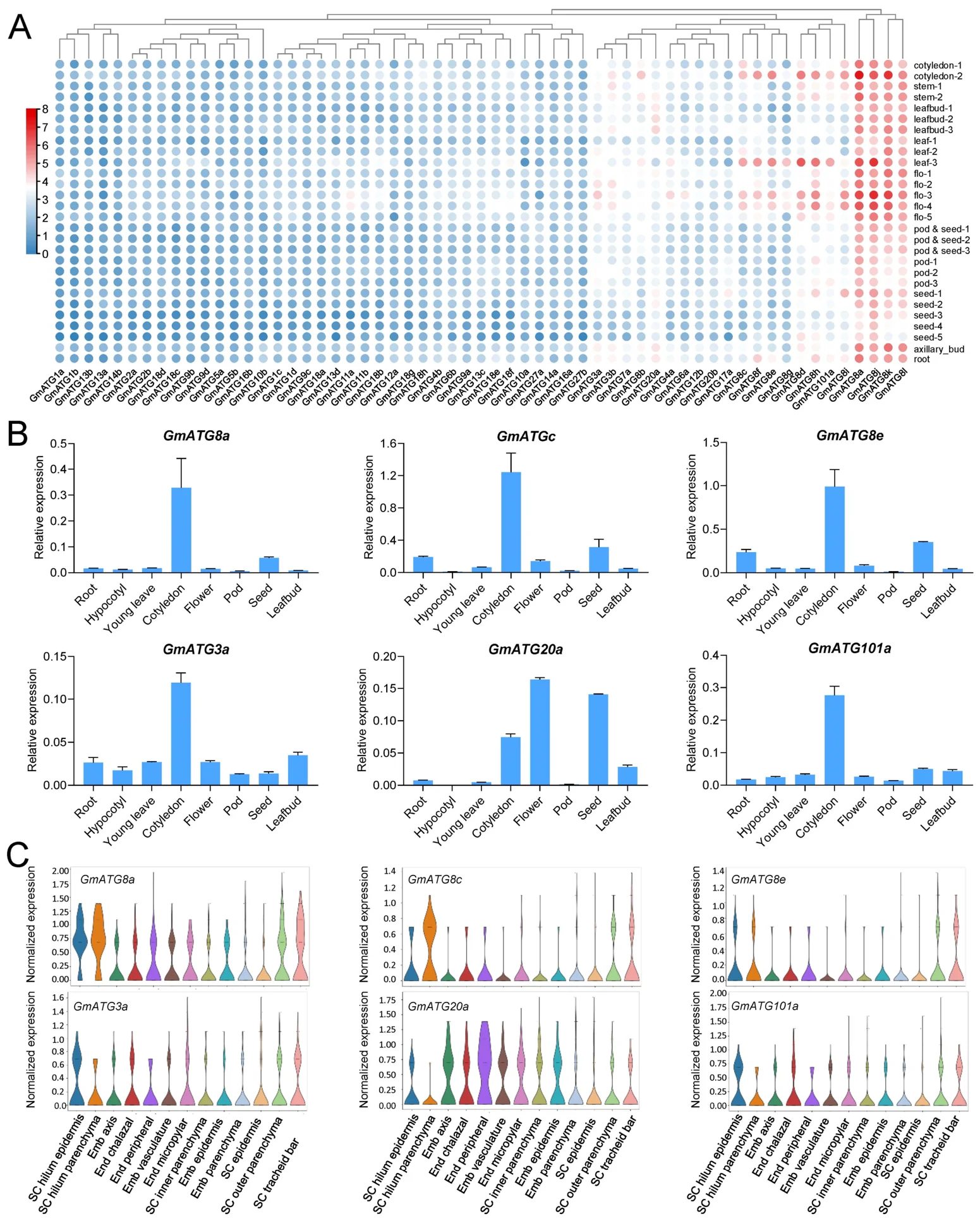
**Tissue expression profiling of *GmATG* genes.** (**A**) Expression profiles of *GmATG* genes across different developmental stages and tissues of soybean based on public RNA-seq data. Samples included emergence stage (VE), cotyledon stage (VC), three-node stage (V3), flowering stage, and various stages of seed development. The heatmap shows Log2-normalized FPKM values. (**B**) RT-qPCR validation of selected *GmATG* gene expression in different tissues. Relative expression levels were calculated using the 2^−ΔΔCt^ method with Tubulin as the internal control. Data are means ± SD of three biological replicates. (**C**) Spatial expression patterns of *GmATG* genes in cotyledon-stage seeds at single-cell resolution. SC: seed coat; Emb: embryo.

To investigate the tissue expression patterns of the *GmATG* gene family, transcriptomic data from the W82 line were analyzed for roots, hypocotyls, cotyledons, leaves, leaf buds, flowers, pods, and seeds based on the SoyOmics database. Heatmap analysis revealed that members of the *GmATG* family exhibited distinct expression patterns (Figure 5A). Multiple members of the *GmATG8* subfamily (*GmATG8a*/*8j*/*8k*/*8l*) showed constitutive high expression across all tissues. Some genes exhibited tissue-specific expression: *GmATG3a* and *GmATG3b* were specifically highly expressed in flowers; *GmATG20a* was most prominently expressed in leaf buds; *GmATG101a* was highly expressed in cotyledons and leaves. Notably, *GmATG8a*/*8h*/*8i*/*8j*/*8k*/*8l* were enriched in seeds. Most other *GmATG* members exhibited low expression levels across all tissues.

To validate the reliability of the RNA-seq data, six *GmATG* genes with distinct tissue expression patterns were selected (*GmATG8a* was highly expressed in all tissues; *GmATG8e*, *GmATG8c*, and *GmATG101a* were enriched in cotyledons, leaves, and flowers; *GmATG3a* was preferentially expressed in flowers; *GmATG20a* was highly expressed in leaf buds) for RT-qPCR analysis (Figure 5B). The results showed that, except for *GmATG20a*, which exhibited high expression in flowers and seeds, the remaining five genes were most highly expressed in cotyledons. We further analyzed the single-cell transcriptomic data from cotyledon-stage seeds of W82 [52] to reveal the spatial expression patterns of the six *GmATG* genes (Figure 5C). The results showed that *GmATG8a* and *GmATG8c* were specifically highly expressed in the hilum epidermis and the inner parenchyma of the seed coat (SC), respectively. *GmATG20a* was specifically enriched in the chalazal endosperm, while *GmATG101* exhibited low-level, uniform expression in the peripheral endosperm. In embryonic tissues, *GmATG3a* exhibited moderate, uniform expression in the embryonic axis. These results indicated that *GmATG* genes form a spatially organized expression network in seeds, with specific members enriched at nutrient transport interfaces (hilum and chalazal endosperm), potentially performing specialized functions in seed development and nutrient translocation.

**Figure 6:**
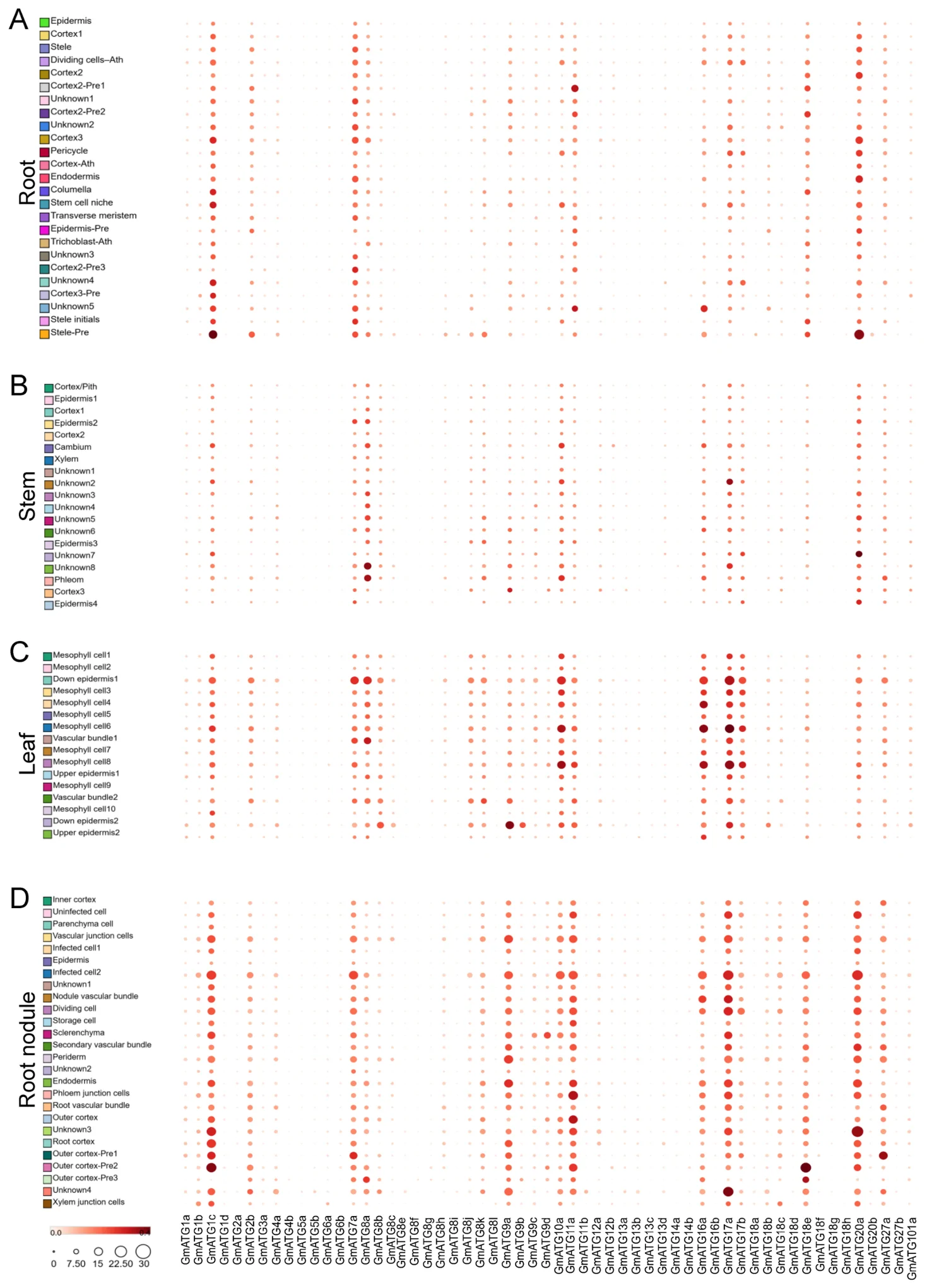
**Single-cell transcriptomic analysis of *GmATG* genes.** Expression patterns of GmATG genes in root (**A**), stem (**B**), leaf (**C**), and root nodule (**D**) tissues in ZH13. Roots, stems, leaves, and shoot apical meristems were collected at the vegetative cotyledon stage; nodules were harvested 5 days after inoculation with Bradyrhizobium USDA-110 at the vegetative cotyledon stage. Color intensity indicates expression level (darker color = higher expression). Circle size represents the percentage of cells expressing the gene among all detected cells in that specific region.

To further elucidate the cellular expression heterogeneity of *GmATG* genes, we analyzed published single-cell transcriptomic data from roots, stems, leaves, and root nodules of ZH13 [51] (Figure 6). The results showed that *GmATG1c* and *GmATG20a* were consistently highly expressed in almost all cell types, with the most significant expression observed in roots and root nodules; *GmATG2b*/*7a*/*8a*/*9a*/*10a*/*11a*/*16a*/*17a*/*18e*/*17b*/*27a* were widely expressed across multiple tissues; whereas subfamily members such as *GmATG5*/*6*/*12*/*13*/*14* exhibited extremely low expression levels in all cell types.

### 3.8. Expression Analysis of the GmATG Family Under Phosphorus Deficiency

**Figure 7:**
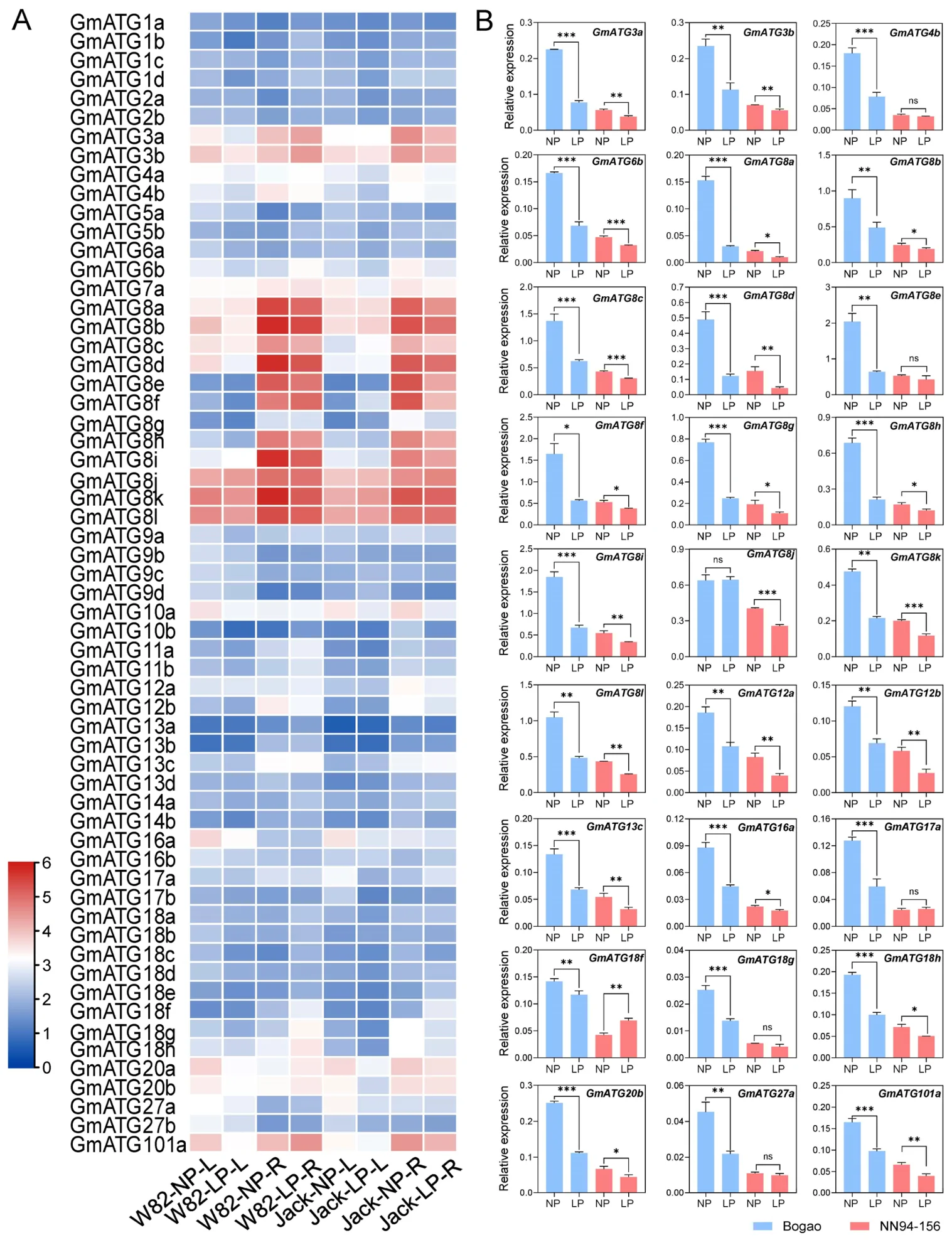
**Expression analysis of *GmATG* genes under low-phosphorus stress.** (**A**) Transcriptomic heatmap of *GmATG* gene expression in roots (R) and leaves (L) of the low-phosphorus-tolerant variety W82 and the sensitive variety Jack under normal-phosphorus (NP, 500 μM Pi) and low-phosphorus (LP, 5 μM Pi) treatments. (**B**) RT-qPCR validation of selected *GmATG* gene expression in roots of the low-phosphorus-tolerant variety NN94-156 and the sensitive variety Bogao under low-phosphorus stress. Relative expression levels were calculated using the 2^−ΔΔCt^ method with Tubulin as the internal control. Data are means ± SD of three biological replicates. * *p* < 0.05, ** *p* < 0.01, *** *p* < 0.001, ns, no significant (Student’s *t*-test). Blue: Bogao; red: NN94-156.

To investigate the response characteristics of the *GmATG* gene family to phosphorus deficiency, we analyzed the gene expression profiles in roots and leaves of the low-phosphorus-tolerant variety W82 and the sensitive variety Jack under low-phosphorus (LP, 5 μM Pi) and normal-phosphorus (NP, 500 μM Pi) treatments based on previous comparative transcriptomic data (Figure 7A). The expression levels of *GmATG8* and *GmATG3* subfamilies were relatively higher than other *GmATG* genes, and *GmATG8b*/*8d*/*8i*/*8k* showed the highest expression level in roots. Almost all *GmATG8* genes showed higher expression level in roots under the NP condition than under the LP condition and in roots of W82 compared to Jack. In roots, *GmATG1*/*2*/*3*/*5*/*6*/*7*/*9*/*10*/*11*/*13*/*14*/*18*/*20*/*27*/*101* subfamilies of W82, and *GmATG2*/*5*/*13*/*17* and parts of *GmATG18* subfamilies of Jack, showed higher expression levels. The high expression and similar expression patterns of the *GmATG8* subfamily in Jack and W82 indicate that the *GmATG8* subfamily may be involved in response in phosphorus deficiency.

To further validate the expression responses of the *GmATG* genes to phosphorus deficiency, based on transcriptomic analysis results, 27 representative members with relatively high expression were selected for RT-qPCR. The LP-tolerant variety Nannong 94-156 (NN94-156) and LP-sensitive variety Bogao were cultured hydroponically under NP and LP conditions for 11 days (Figure 7B). The results showed that the expression levels of *GmATG* genes were higher in Bogao than NN94-156 under both NP and LP conditions. In addition, most genes showed significantly higher expression level under the NP condition than under the LP condition, in both NN94-156 and Bogao, except for *GmATG18f* in NN94-156 that was more highly expressed under the LP condition and *GmATG4b*/*8e*/*17a*/*18g*/*27a*/*27b* in NN94-156 and *GmATG8j* in Bogao that showed no difference between NP and LP conditions. In addition, *GmATG8b-8l* showed higher expression levels, and 16 other *GmATG* genes showed lower expression levels. The significantly different expression levels under NP and LP treatments suggest that *GmATG* genes, especially *GmATG8b-8l*, might be involved in soybean adaptation to phosphorus deficiency, and *GmATG18f* may have a specific function in response to phosphorus deficiency according to its genotype-specific expression.

### 3.9. Expression Analysis of GmATG Family Under Salt Stress

**Figure 8:**
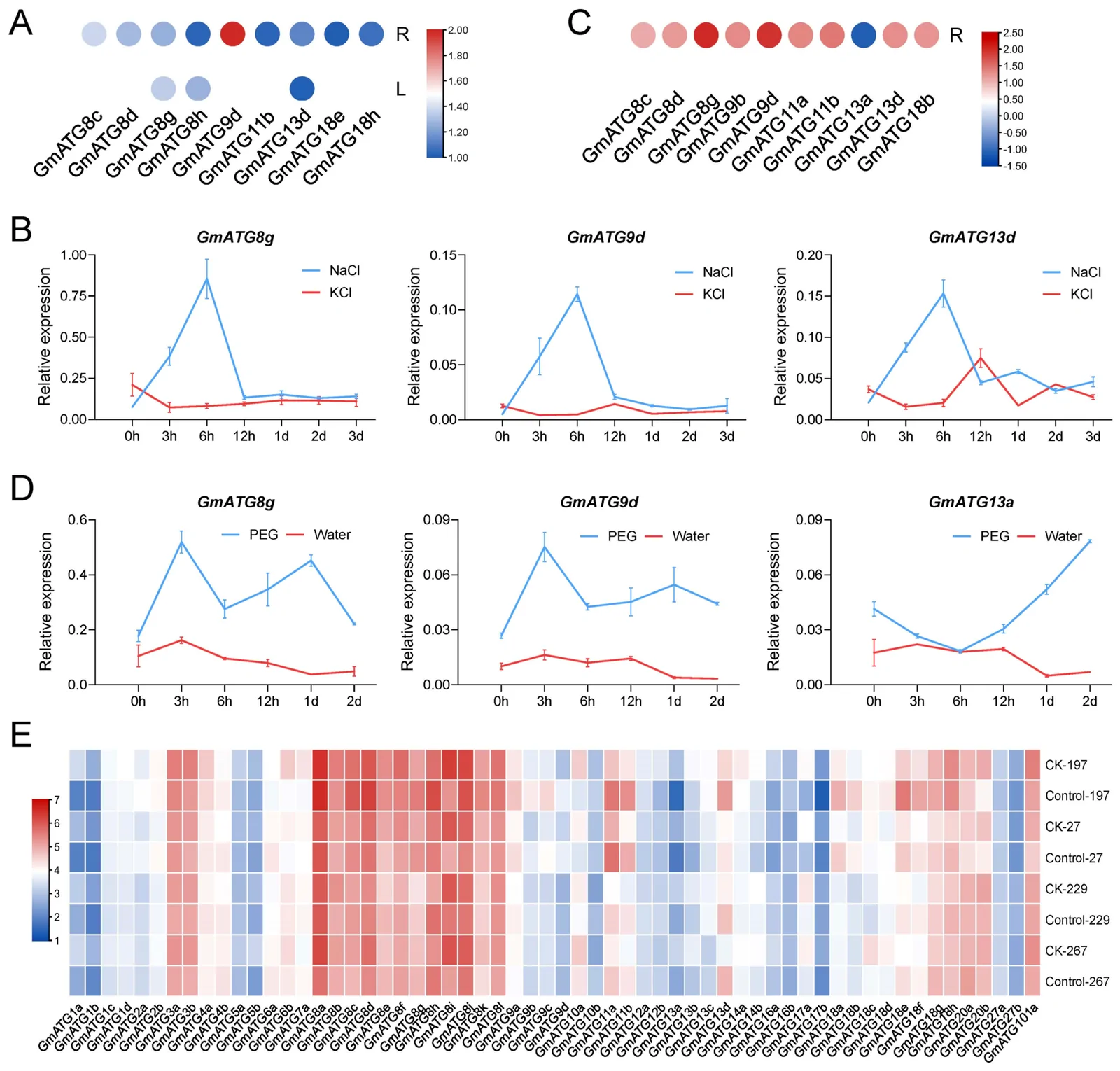
**Expression analysis of *GmATG* genes under stress.** (**A**) Transcriptomic heatmap of *GmATG* gene expression in roots and leaves under salt stress. (**B**) RT-qPCR validation of the expression dynamics of *GmATG8g*, *GmATG9d*, and *GmATG13d* in roots under salt stress. Treatment times: 0 h, 3 h, 6 h, 12 h, 1 d, 2 d, 3 d. Relative expression levels were calculated using the 2^−ΔΔCt^ method with Tubulin as the internal control. Data are means ± SD of three biological replicates. (**C**) Transcriptomic heatmap of *GmATG* gene expression in roots under drought stress. (**D**) RT-qPCR validation of the expression dynamics of *GmATG8g*, *GmATG9d*, and *GmATG13a* in roots under drought stress. Treatment times: 0 h, 3 h, 6 h, 12 h, 1 d, 2 d. (**E**) Heatmap of GmATG gene expression in susceptible lines (H197, H027) and resistant lines (H229, H267) under *Phomopsis longicolla* infection.

To evaluate the potential function of *GmATG* genes in response to salt stress, we analyzed the expression levels of *GmATG* genes based on transcriptomic data. The transcriptomic heatmap showed that the expression of *GmATG9d*, *GmATG8c*/*8d*/*8g*, and *GmATG8h*/*11b*/*13d* in roots exhibited the highest, moderate, and lower upregulation under salt stress, respectively (Figure 8A). In leaves, *GmATG8g*/*h* and *GmATG13d* exhibited relatively high and low upregulation, respectively. Based on the fold changes and significance levels in the transcriptomic heatmap, three key genes (*GmATG8g*/*9d*/*13d*) were selected for RT-qPCR validation with root samples of W82 treated with 120 mM NaCl at 0 h, 3 h, 6 h, 12 h, 1 d, 2 d, and 3 d. The results showed that the expression of *GmATG8g*/*9d*/*13d* followed a “rise-then-fall” pattern: expression gradually increased from 0 h to 6 h of treatment and subsequently declined from 6 h to 12 h (Figure 8B). In contrast, under control treatment (equimolar KCl), the expression levels of these genes remained low with no significant fluctuations. RT-qPCR validation results generally agreed with the transcriptomic data, but the upregulation of *GmATG8g* was the most significant.

### 3.10. Expression Analysis of GmATG Family Under Drought Stress

To evaluate the potential function of *GmATG* genes in response to drought stress, we first analyzed the expression levels of *GmATG* genes based on the transcriptomic data in roots of W82 under drought treatment for 6 d. The transcriptomic heatmap showed that *GmATG8g*/*9d* were the most significantly upregulated, *GmATG8c*/*8d*/*9b*/*11*/*13d*/*18b* were upregulated to varying degrees, whereas *GmATG13a* was downregulated, indicating that the *GmATG* genes exhibit differential expression patterns under drought stress (Figure 8C).

Based on the differential expression patterns and trends observed in the transcriptomic heatmap, three key genes (*GmATG8g*/*9d*/*13a*) were selected for validation via RT-qPCR. RT-qPCR analysis was performed on root samples of W82 treated with 20% PEG-6000 at 0 h, 3 h, 6 h, 12 h, 1 d, and 2 d. The results showed that *GmATG8g* and *GmATG9d* exhibited an early rapid induction pattern under drought stress: *GmATG8g* exhibited two expression peaks at 3 h and 24 h (relative expression levels of approximately 0.5 and 0.45, respectively) under PEG treatment; *GmATG9d* reached a peak at 3 h under PEG treatment and then gradually declined. In contrast, *GmATG13a* exhibited a sustained upward trend after 6 h of PEG treatment, reaching its highest value (relative expression of approximately 0.08) after 2 days of treatment (Figure 8D). Those results indicate that *GmATG8g* and *GmATG9d* may be involved in rapid drought response and *GmATG13a* may be involved in late drought response.

### 3.11. Transcriptional Responses of GmATG Genes to Phomopsis Seed Decay

To evaluate the potential function of *GmATG* genes in resistance to Phomopsis seed decay, we analyzed the transcriptomic data of four soybean genotypes (susceptible lines H197 and H027; resistant lines H229 and H267) 1 d after inoculation with *P. longicolla* (unpublished). Heatmap analysis revealed that *GmATG* genes exhibited diverse response patterns to *P. longicolla* infection (Figure 8E): *GmATG8* showed the highest expression, and *GmATG3*/*4*/*6*/*11*/*13d*/*18g*/*18h*/*20*/*101a* showed relatively high expression in both susceptible and resistant materials. *GmATG4*/*8h*/*9a*/*11*/*13d*/*18d*/*18e*/*18f*/*18g*/*18h* were upregulated after infection in both susceptible and resistant lines, *GmATG1a*/*1b*/*2b*/*6b*/*8i*/*10a*/*13a*/*13b*/*14*/*16a*/*17*/*20b*/*101a* were downregulated after infection in both susceptible and resistant lines, *GmATG2a*/*7a*/*9b*/*9c*/*9d*/*18a*/*18b* were upregulated after infection in only susceptible lines, and *GmATG7a* was downregulated in susceptible lines and upregulated in resistant lines after infection. The relatively high expression and different expression patterns in susceptible and resistant soybean lines of *GmATG7a*/*8h*/*8i*/*11*/*13d*/*18e*/*18f* after infection suggest that these *GmATG* genes may be involved in soybean immunity.

## 4. Discussion

### 4.1. Evolutionary Expansion and Fragment Duplication Characteristics of GmATG Family

The size of the *ATG* gene family varies significantly among species. In this study, we identified 60 *GmATG* genes (Figure 1) belonging to 20 subfamilies, which is more than yeast, Arabidopsis, *Medicago truncatula*, and rice [9,58,59,60,61]. Fragment duplication is the primary mechanism driving the expansion of the *GmATG* family (33 pairs of duplicated fragments), which is consistent with the WGD-driven gene family expansion pattern as reported in a comparative analysis of autophagy families in *Phaseolus vulgaris*, *M. truncatula*, and soybean [62]. There are significant differences in the expansion patterns among different *ATG* subfamilies: *ATG7* and *ATG101* remain single-copy, while the *ATG8* and *ATG18* families have expanded significantly (Table S2), which is consistent with the characteristic of Arabidopsis, *M. truncatula*, and rice, where the core autophagy gene (*ATG7*) is single-copy, and the substrate recognition and membrane recruitment modules (*ATG8* and *ATG18*) are multi-copy [6,7,9,61], suggesting that genes encoding the core catalytic enzymes are constrained by purifying selection to maintain dose sensitivity, whereas families involved in substrate recognition and membrane recruitment gain functional diversity through copy number expansion. These findings suggest that WGD-derived duplicates are preferentially retained for autophagy-related functions across legumes, likely reflecting a conserved requirement for spatiotemporal expression diversification under varying environmental and symbiotic conditions. As autophagy components, the soybean *ATG8* family has expanded to 12 members (*GmATG8a-l*), which was higher than other legumes, such as *M. truncatula* (nine members) and *P. vulgaris* (eight members) [61,62], providing an expanded genetic reservoir for substrate recognition in selective autophagy. In addition, we also found that *GmATG8d* and *GmATG10b* were cultivar W82 specific, which were not identified in 15 other cultivars, nine landraces, and four wild soybeans, indicating that those two genes may have been formed during modern breeding.

### 4.2. Expression Characteristics and Functional Differentiation of GmATG Genes in Nutrient Cycling

Tissue expression profiles showed that *GmATG8* subfamily genes were highly expressed in leaf, flower, and cotyledon, and *GmATG8a*//*8k*/*8l* were highly expressed in all tissues, while other *GmATG* genes were expressed at lower levels (Figure 5A,B). Single-cell transcriptomic analysis showed that *GmATG8a* and *GmATG8c* were specifically highly expressed in the hilum epidermis and the inner parenchyma of the seed coat, respectively. As the nutritional interface connecting the seed to the parent plant, the functional heterogeneity of the cell populations in the hilum may be related to nutrient transport efficiency. Considering that *M. truncatula ATG8s* were highly induced during seed development [61], ATGs may be involved in legume reproduction. Collectively, the seed-enriched and cell-type-specific expression of *GmATG8* members suggests they achieve functional specialization through precise spatiotemporal expression patterns, which may be an adaptive mechanism to meet the complex demands of seed development and storage compound accumulation in leguminous crops.

Under carbon and nitrogen starvation, autophagy participates in the degradation of starch, chloroplast protein and even the whole chloroplast for the maintenance of free amino acid pools and the supply of respiratory substrates [63]. Single-cell transcriptomic analysis further revealed spatial expression advantages of *GmATG7a*/*8a*/*10a*/*16a*/*17a* in mesophyll cells of leaves (Figure 6C), which are responsible for photosynthesis and nutrient storage. Their homologous *Arabidopsis ATG5* genes influenced photosynthetic efficiency in leaves [64] and apple *MdATG10* overexpression mitigated photosynthetic damage in response to cadmium stress [65], indicating that these five *GmATGs* may be involved in photosynthesis under carbon starvation and stress. Additionally, single-cell transcriptomic analysis further revealed spatial expression advantages of *GmATG1c*/*9a*/*11a*/*17a*/*20a* in root nodules and *GmATG1c*/*7a*/*20a* in roots, their homologous genes *ATG1* and *ATG8* in *M. truncatula* were involved in the mitophagy that controls symbiotic nitrogen fixation [66], indicating that these six GmATGs may be involved in root nitrogen absorption and symbiotic nitrogen fixation. Phosphorus deficiency can induce autophagy [67], and *Arabidopsis ATG* expression was upregulated under LP stress [63]. While our expression analysis showed that most *GmATG* genes were downregulated under LP stress in both LP-sensitive and -resistant genotypes, especially the *GmATG8* subfamily genes that showed the highest expression level, only *GmATG18f* was upregulated under LP stress in the LP-resistant genotype (Figure 7B), and these results indicate that *GmATG* may be involved in maintaining phosphorus homeostasis in a different way.

### 4.3. Expression Characteristics and Functional Differentiation of GmATG Genes Under Stress

Under *P. longicolla* infection, multiple *GmATG* members exhibit differential expression, as *GmATG4*/*8h*/*9a*/*11*/*13d*/*18d*/*18e*/*18f*/*18g*/*18h* were upregulated after infection in both susceptible and resistant lines, consistent with the understanding that autophagy is activated during pathogen infection [26]: *Arabidopsis ATG3*, *ATG8a*, *ATG8h* and *ATG8i*, and *ATG18a* positively regulate resistance to turnip mosaic virus (TuMV) [68], CMV-Δ2b and CMV-2aTΔ2b [69], powdery mildew [70], and the necrotrophic pathogen *Botrytis cinerea* [71], respectively; rice *OsATG8* enhances immunity [72]; wheat *TaATG8g* positively regulates resistance to powdery mildew [73]; tomato *ATG4*/*6*/*8a*/*8b*/*7*/*10*/*13d* positively regulate resistance to nematodes [74]. *GmATG1a*/*1b*/*2b*/*6b*/*8i*/*10a*/*13a*/*13b*/*14*/*16a*/*17*/*20b*/*101a* were downregulated after infection in both susceptible and resistant lines, and their homologous *OsATG1* negatively regulates rice immunity [72], *Arabidopsis ATG8i* enhanced the resistance to biotrophic bacterial and fungal pathogens but compromised the resistance to a toxin secreted from a necrotropic fungal pathogen [75], and apple *MdATG8i* promotes resistance to necrotropic fungus *Valsa mali* [76]. In addition, *GmATG2a*/*9b*/*9c*/*9d*/*18a*/*18b* were upregulated after infection in only susceptible lines, and *GmATG2* and *GmATG7* have been proved to negatively regulate soybean resistance to *Pseudomonas syringae* pv. *Glycinea* [34,35], indicating that these *GmATG*s may be involved in the negative regulation of soybean resistance to *P. longicolla*. Their upregulated expression may be induced by pathogens to manipulate host autophagy to facilitate infection, as it has been reported that citrus tristeza virus (CTV) p20 could interact with ATG8 to activate autophagic degradation of plant-specific RNA-binding protein SGS3 and promote CTV infection [77]; the capsid protein P10 of black-streaked dwarf virus (RBSDV) could promote the degradation of OsFAD7 mediated by OsATG8, thereby promoting the infection of RBSDV [78]. Furthermore, *GmATG18f* was induced after infection and phosphorus deficiency, indicating that *GmATG18f* was involved in both soybean immunity and phosphorus deficiency. However, *GmATG4*/*8h*/*9a*/*11*/*13d*/*18d*/*18e*/*18g*/*18h* were induced after infection but downregulated under phosphorus deficiency, and the opposite expression pattern may be because the two stresses were performed under a nonmycorrhizal condition, and nonmycorrhizal phosphorus-acquisition strategies enhance plant pathogen susceptibility [79].

In soybean, *GmATG8c*/*8d*/*8g*/*8h*/*9d*/*11b*/*13d*/*18e*/*18h* were upregulated under salt stress, and *GmATG8g*/*9d*/*13d* exhibited a transient increase followed by a decreased expression pattern, peaking at 6 h after treatment and then declining. Their homologous gene in rice, *OsATG8d*, improves growth under salt stress [15]. Under drought stress, *GmATG8c*/*8d*/*8g*/*9b*/*9d*/*11*/*13d*/*18b* were upregulated, and *GmATG8g* and *GmATG9d* exhibited an early rapid induction pattern. Their homologous genes, maize *ZmATG8a*/*13a*/*18a*, were upregulated by ZmNBR1 to promote the autophagic degradation of ZmBRI [80], tomato *ATG18f* was activated by HsfA1a to induce autophagy to degrade drought-induced insoluble proteins [81], and *M. truncatula* ATG8 interacts with MtCAS31 to promote autophagic degradation of drought tolerance negative regulator MtPIP2;7 [82], then enhance thermotolerance. *GmATG8c*/*8d*/*8g*/*9d*/*11b*/*13d* were upregulated under both salt and drought stress, and the rapid induction of *GmATG8g*/*9d* is consistent with studies showing rapid activation of autophagy in *Arabidopsis* under osmotic stress [83]; whereas the sustained upregulation of *GmATG13a* may reflect its distinct role in adaptation to long-term stress. It is worth noting that all the candidate *GmATG* genes that may be involved in soybean’s response to stresses were identified through the analysis of changes in expression patterns under various stresses, but their specific functions still need further verification.

## 5. Conclusions

This study systematically characterized the *GmATG* gene family, identifying 60 *GmATG* genes distributed across 20 subfamilies. Fragment duplication (33 pairs) was the primary driver of family expansion, with the *ATG8* and *ATG18* subfamilies showing significant expansion while the core genes *ATG7* and *ATG101* remained single-copy. Pan-genomic analysis further revealed extensive copy number variation in the *ATG18* family, providing a genetic basis for functional differentiation. Stress-induced *GmATG*s were selected and supposed to participate in soybean response to stress: most *GmATG* genes showed reduced expression under phosphorus deficiency, only *GmATG18f* was significantly upregulated in the low-phosphorus-tolerant variety NN94-156; *GmATG8g*, *GmATG9d*, and *GmATG13d* were induced within 6 h of salt stress; *GmATG8g*, *GmATG9d*, and *GmATG13a* were induced under drought stress; the relatively high expression and different expression patterns of *GmATG7a*/*8h*/*8i*/*11*/*13d*/*18e*/*18f* suggest they may be involved in soybean resistance to Phomopsis stem rot. Notably, *GmATG18f* was induced under both phosphorus deficiency and biotic stress, and *GmATG8g/9d* was induced under both salt and drought tolerance, suggesting a potential role in response to stress. The specific functions of these candidate *GmATG* genes will be verified in future.

## Data Availability

The datasets used and/or analyzed during the current study are available from the corresponding author on reasonable request. Some of the data are shown in the Supplementary Materials.

## Supplementary Materials

The following supporting information can be downloaded at: https://www.mdpi.com/article/10.3390/genes17090996/s1. Table S1. Amino acid sequence of ATG genes in soybean, *Arabidopsis thaliana*, and rice. Table S2. The characteristics of members of *GmATG* gene family. Table S3. *GmATG* genes with their orthologs in *Arabidopsis thaliana* and *Glycine soja*. Table S4. *ATG* genes in 29 soybean accessions. Table S5. Number of *ATG* genes in 29 soybean accessions. Table S6. The intron phase of GmATG genes. Table S7. Expression profiles of *GmATG* genes in multiple tissues throughout various developmental stages. Table S8. *GmATG* expression under different phosphate level treatments. Table S9. Expression profiles of *GmATG* genes in soybean stems in response to *Phomopsis longicolla* infection. Table S10. *GmATG* expression under different drought treatments. Table S11. *GmATG* expression under different Na+ level treatments. Table S12. Primers used in this study for RT-qPCR.
